# RBGO - First Impact Factor: 1.2

**DOI:** 10.1055/s-0043-1772497

**Published:** 2023-08-18

**Authors:** Marcos Felipe Silva de Sá

**Affiliations:** 1 Editor RBGO

RBGO - Revista Brasileira de Ginecologia e Obstetrícia is proud to announce the achievement of its first Impact Factor. This accomplishment supports RBGO's position as the leading Gynecology and Obstetrics journal in Latin America.

Having a high-quality ObGyn journal published in Brazil has been the goal of Brazilian researchers so that the high-quality science produced here could be increasingly disseminated internationally. As a Society that proposes to support the training and education of professionals in Gynecology and Obstetrics, the Brazilian Federation of the Association of Gynecology and Obstetrics – FEBRASGO- also understood this need and did its utmost to offer our Brazilian researchers, residents, postgraduate students, and professionals a high-level and internationally competitive journal.

**Fig. 1:**
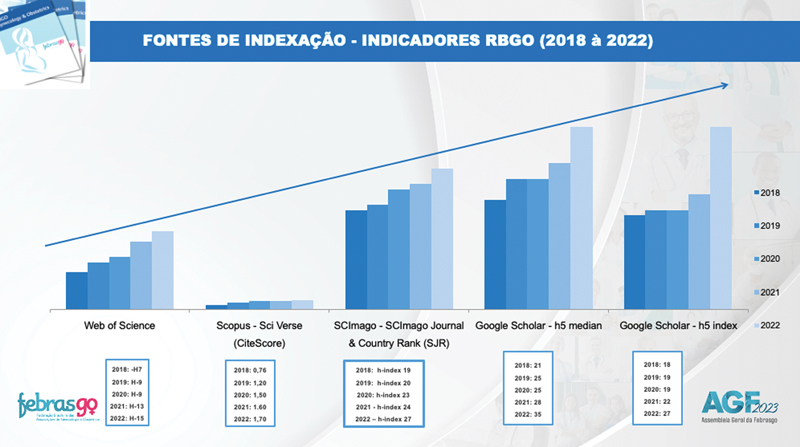
RBGO performance in the previous five years according to international indicators.

In the last seven years, RBGO has been completely reformulated: an English edition was made available, it became an “open access” journal, its board of associate editors was restructured, it is now indexed in the main databases of international scientific literature and the periodicity was regularized. All these improvements made it an international journal that is offered cost free to the authors. The indicators below ( Fig. 1 ) demonstrate the positive effect of the measures taken by RBGO. The main goal was to achieve the Impact Factor which has a special meaning since it places the journal on the list of the widely disseminated journals internationally.

Finally, this June, RBGO was awarded an Impact Factor of 1.2 by Clarivates after a rigorous evaluation during the past 5 years. We take advantage of this historic moment to invite all authors to join our academic community! There are many benefits of publishing in RBGO which, from now on, gains greater importance and competitiveness in the dissemination of works published in the journal. Our great challenge in the future is to maintain this “status” with a pretentious desire to ascend.

We thank the authors who submitted their manuscripts to RBGO, the Associate Editors and “anonymous” reviewers, the Editorial Office team and Febrasgo collaborators. A special thanks to the Executive Board of Febrasgo, who believed in the proposal of the NEW RBGO and offered their unconditional support to the Editors.

